# Supporting the analysis of ontology evolution processes through the combination of static and dynamic scaling functions in OQuaRE

**DOI:** 10.1186/s13326-016-0091-z

**Published:** 2016-10-17

**Authors:** Astrid Duque-Ramos, Manuel Quesada-Martínez, Miguela Iniesta-Moreno, Jesualdo Tomás Fernández-Breis, Robert Stevens

**Affiliations:** 1 Universidad de Murcia, IMIB-Arrixaca, Campus de Espinardo, Murcia, 30071 Spain; 2 School of Computer Science, University of Manchester, Oxford Road, Manchester, M13 9PL UK

**Keywords:** Ontology quality, Ontology metrics, Oquare, Ontology repositories

## Abstract

**Background:**

The biomedical community has now developed a significant number of ontologies. The curation of biomedical ontologies is a complex task and biomedical ontologies evolve rapidly, so new versions are regularly and frequently published in ontology repositories. This has the implication of there being a high number of ontology versions over a short time span. Given this level of activity, ontology designers need to be supported in the effective management of the evolution of biomedical ontologies as the different changes may affect the engineering and quality of the ontology. This is why there is a need for methods that contribute to the analysis of the effects of changes and evolution of ontologies.

**Results:**

In this paper we approach this issue from the ontology quality perspective. In previous work we have developed an ontology evaluation framework based on quantitative metrics, called OQuaRE. Here, OQuaRE is used as a core component in a method that enables the analysis of the different versions of biomedical ontologies using the quality dimensions included in OQuaRE. Moreover, we describe and use two scales for evaluating the changes between the versions of a given ontology. The first one is the static scale used in OQuaRE and the second one is a new, dynamic scale, based on the observed values of the quality metrics of a corpus defined by all the versions of a given ontology (life-cycle). In this work we explain how OQuaRE can be adapted for understanding the evolution of ontologies. Its use has been illustrated with the ontology of bioinformatics operations, types of data, formats, and topics (EDAM).

**Conclusions:**

The two scales included in OQuaRE provide complementary information about the evolution of the ontologies. The application of the static scale, which is the original OQuaRE scale, to the versions of the EDAM ontology reveals a design based on good ontological engineering principles. The application of the dynamic scale has enabled a more detailed analysis of the evolution of the ontology, measured through differences between versions. The statistics of change based on the OQuaRE *quality scores* make possible to identify key versions where some changes in the engineering of the ontology triggered a change from the OQuaRE quality perspective. In the case of the EDAM, this study let us to identify that the fifth version of the ontology has the largest impact in the quality metrics of the ontology, when comparative analyses between the pairs of consecutive versions are performed.

## Background

In recent years the biomedical community has increased its effort in the development of ontologies and this is likely to continue [1]. Ontology developers tend to publish their ontologies on the Web and they are accessible from different sources. BioPortal [2], for instance, contains more than 500 ontologies at the time of writing and new content is published frequently. BioPortal enables updates by user submissions of new versions, which are accessible via web browsers and through web services [2].

The curation of ontologies is often a complex task because of their high level of activity and rapid evolution [3]. For this reason, the number of versions of an ontology may grow rapidly. The evolution process turns the development of an ontology into a dynamic process. Each of the different versions of an ontology constitutes a snapshot of this process. The analysis of versions was introduced by [4], in which ontology versioning was defined as the ability to handle changes in ontologies by creating and managing different variants of it and which pointed out the importance of highlighting differences between versions. Later, [5] claimed that a versioning system for ontologies must compare and present structural changes rather than changes in text representation or source files. They described a version-comparison algorithm that produces a structural difference between ontologies, which was presented to users through an interface for analysing them [6]. As mentioned in [7], there is no distinction between versioning and evolution in ontologies since both account for the management of changes in ontologies.

If we approach ontology changes from a logical perspective those changes are usually materialised by modifying the axioms of a given ontology. Those modifications may imply the addition or removal of classes, properties, individuals or constraints, as well as modifying the characteristics, domains and ranges of properties. Such number and types of changes have been the inputs for different approaches that have tried to understand the evolution of ontologies:

- Bubastis [3, 8] analysed the degree of activity in biomedical ontologies by considering 5 major types of ontology changes between two consecutive versions: added or removed axioms to an existing named class (NC), NCs added, NCs made obsolete and edited annotation properties.
- Copeland et al. 2013 [9] focused on changes in asserted and inferred axioms taking into account reasoning capabilities in ontologies [10].
- In [11] a web application providing an interactive and user-friendly interface to identify (un)stable regions in large life science ontologies is proposed. A method that computes change intensities for regions based on changes between several succeeding versions of an ontology within a specific time interval is used.

It makes sense to think that the changes made to an ontology across its different versions should have an impact on its quality. In addition, assuming that the changes in an ontology should have a positive impact on the quality of that ontology is also reasonable. In this context, the main contribution of this work is to the study of the evolution of ontologies from the perspective of ontology quality, since, to the best of our knowledge, this aspect has not been significantly researched to date. The analysis of quality in ontologies has been addressed in different ways in the ontology evaluation community, such as in the following works:

- Gangemi et al. 2006 [12] approached it as a diagnostic task based on ontology descriptions, using three categories of criteria (structural, functional and usability profiling).
- Rogers 2006 [13] proposed an approach using four qualitative criteria (philosophical rigour, ontological commitment, content correctness, and fitness for a purpose).
- Yao et al. 2005, Tartir and Arpinar 2007 [14, 15] presented metrics for evaluating structural properties in the ontology.
- Duque-Ramos et al. 2011 [16] proposed OQuaRE, which adapts the SQuaRE standard for software quality evaluation for defining a qualitative and quantitative ontology quality framework.

Our proposal is based on the OQuaRE Framework [16], which is a qualitative and quantitative ontology quality framework. The OQuaRE is based on the standard for Software product Quality ISO/IEC 25000:2005 (SQuaRE) [17]. The application of SQuaRE (1) provides a comprehensive specification and evaluation model for software product quality; (2) makes quality evaluation reproducible and objective, based on observations; and (3) allows for a common language for specifying user requirements that is understandable by users, developers and evaluators. All these properties are desirable for an ontology quality evaluation approach. Ontologies, conceived as a special kind of information object or computational artifact [18], have a series of shared notions with Object Oriented Design [19]. For example, the existence of classes, individuals and properties can be exploited to adapt Object Oriented Programming metrics to ontologies. This leads us to believe that the principles of SQuaRE can be adapted to ontologies. Thus, the main goal of OQuaRE is to provide an objective, standardised framework for ontology quality evaluation, applicable to different ontology evaluation scenarios, in which the ontologies are evaluated as final products. For this, OQuaRE includes a generic scaling function that transform metrics values into quality scores.

In this work, we adapt OQuaRE for the purpose of measuring the impact of the evolution of ontologies in their quality. In [20], we described how OQuaRE could be used to evaluate the quality of the different versions of the ontology of Bioinformatics operations, types of data, formats, and topics (EDAM) [21]. The standard quality model and metrics defined in OQuaRE were used and the method was able to detect changes in the measured quality of the different versions of the EDAM. The present work is an extension of [20], presenting methodological evolution and progress. First, we further formalise the method to measure differences between versions of the same ontology based on the OQuaRE performance. Second, we take advantage of such a formalisation for proposing a more sensitive scaling function to be able to detect small differences between consecutive versions of an ontology from the quality metrics perspective. This will let OQuaRE to have two different scaling functions; one for evaluating ontologies and final products and one for evaluating the different versions of a given ontology. The latter is used as feedback to adjust or define new profiles of the static scale. Third, a statistical analysis of the relation of changes in OQuaRE with the profile of activity of the ontology is included. This extension of the OQuaRE framework will allow a better understanding of the evolution of ontologies from a quality perspective and will contribute to demonstrating how ontology quality methods can be used to study ontology evolution.

## Methods

### OQuaRE

OQuaRE is adapted from SQuaRE [17]. SQuaRE defines a quality model and the process for software product evaluation through five divisions: Quality Model, Quality Measurement, Quality Requirements, Quality Evaluation and Quality Management. First, *quality requirements* are identified. Second, the requirements are measured using a *quality model*, which is quantified through *quality metrics*. These three divisions are used by the *quality evaluation* division, which is managed by the *quality management* division. The usage of SQuaRE requires the definition of these five divisions. OQuaRE defines all the elements required for ontology evaluation: evaluation support, evaluation process and metrics. OQuaRE structures the evaluation of the quality of an ontology using the four levels proposed by SQuaRE: quality requirements, quality characteristics, subcharacteristics and metrics. OQuaRE uses the six quality characteristics proposed by SQuaRE for measuring quality: functional adequacy, reliability, operability, maintainability, compatibility, and transferability. Besides, OQuaRE defines a new characteristic, ‘structural’, which accounts for the quality of the structure of the ontology (see Table 1). Each quality characteristic has a set of associated quality subcharacteristics, which are measured through quality metrics. The quality metrics are the units of measurement of quality evaluation. The current version of OQuaRE has 49 *subcharacteristics* and 14 *metrics*. Some OQuaRE subcharacteristics are reused and adapted from SQuaRE, but some others are specific to ontology evaluation. For example, the functional adequacy subcharacteristics are extracted from the intended uses for ontologies identified in [22]. Following a bottom-up approach, *OQuaRE metrics* are combined in order to compose the subcharacteristics, and the subcharacteristics are grouped by the characteristics. Tables 2 and 3 describe respectively how the 14 *OQuaRE* metrics are calculated and how some of the metrics are associated with the *subcharacteristics*. We have not included all of them for simplicity, but they are available at [16, 23].

**Table 1:** OQuaRE characteristics and subcharacteristics used in our method

Characteristic	Description	Associated subcharacteristics
Structural	Formal and semantic relevant ontological properties that account for: the correct use of formal properties, clarity of cognitive distinctions and appropriate use of ontology modelling primitives and principles	“formalisation”, “formal relations support”, “redundancy”, “consistency”, “tangledness”, “cohesion”
Functional Adequacy	Capability of the ontologies to be deployed fulfilling functional requirements, that is, the appropriateness for its intended purpose according to state-of-the art literature [22]	“reference ontology”, “controlled vocabulary”, “schema and value reconciliation”, “consistent search and query”, “knowledge acquisition”, “clustering and similarity”, “indexing and linking”, “results representation”, “text analysis”, “guidance and decision trees” and “knowledge reuse and inferencing”
Reliability	Capability of an ontology to maintain its level of performance under stated conditions for a given period of time	“recoverability” and “availability”
Operability	Effort needed for the ontology use. Individual assessment of such use, by a stated or implied set of users	“learnability”
Compatibility	Capability of two or more ontologies to exchange information and/or to perform their required functions while sharing a hardware/software environment	“replaceability”
Maintainability	Capability of ontologies to be modified for changes in environments, in requirements or in functional specifications	“modularity”, “reusability”, “analysability”, “changeability”, “modification stability” and “testability”
Transferability	Degree to which the ontology can be transferred from one environment (e.g., operating system) to another	“adaptability”

**Table 2:** OQuaRE metrics and a brief description of how we calculate them

OQuaRE metric	Description
ANOnto	Mean number of annotation properties per class
AROnto	Number of restrictions of the ontology per classes
CBOnto	Number of superclasses divided by the number of class
	minus the subclasses of Thing
CROnto	Mean number of individuals per class
DITOnto	Length of the largest path from Thing to a leaf class
INROnto	Mean number of subclasses per class
NACOnto	Mean number of superclasses per leaf class
NOCOnto	Mean number of the direct subclasses per class minus
	the subclasses of Thing
NOMOnto	Mean number of object and data property usages
	per class
LCOMOnto	Mean length of all the paths from leaf classes to Thing
RFCOnto	Number of usages of object and data properties and
	superclasses divided by the number of classes minus
	the subclasses of Thing
RROnto	Number of usages of object and data properties divided
	by the number of subclassof relationships and properties
TMOnto	Mean number of classes with more than 1 direct ancestor
WMCOnto	Mean number of properties and relationships per class

**Table 3:** Summary of the associations between the characteristics, subcharacteristics and the associated metrics

OQuaRE characteristic	OQuaRE subcharecteristic	OQuaRE metric
Structural	Formal relations support	RROnto
	Tangledness	TMOnto
	Cohesion	LCOMOnto
	…	…
Functional adequacy	Controlled vocabulary	ANOnto
	Inference	RROnto, CROnto
	Consistent search and query	ANOnto, RROnto, AROnto, INROnto
	Knowledge acquisition and representation	ANOnto, RROnto, NOMOnto
	…	…
Maintainability	Modularity	WMCOnto, CBOOnto
	Analysability	WMCOnto, DITOnto, RFCOnto, NOMOnto, LCOMOnto, CBOOnto
	Modification stability	WMCOnto NOCOnto RFCOnto LCOMOnto CBOOnto
	…	…
Reliability	Recoverability	WMCOnto, DITOnto, NOMOnto, LCOMOnto,
	Availability	LCOMOnto
	…	…
Operability	Learnability	WMCOnto, LCOMOnto, RFCOnto, NOMOnto, CBOnto, NOCOnto
	…	…

The associations of the reminding 36 subcharacteristics with metrics can be found at http://miuras.inf.um.es/oquarewiki

The evaluation of an ontology comprises a score for those requirements measured through the *quality model*. OQuaRE metrics reuse and adapt a set of well known metrics from both ontology evaluation and software engineering communities [14, 22, 24]. The quality metrics provide quantitative values in different ranges, which are called raw quality metrics values. OQuaRE applies a scaling method recommended in SQuaRE that assigns values in the range [1,5] (5 levels):

- 1 - “Not Acceptable”
- 2 - “Not Acceptable - Improvement Required”
- 3 - “Minimally Acceptable”
- 4 - “Acceptable”
- 5 - “Exceeds Requirements”

**Fig. 1:**
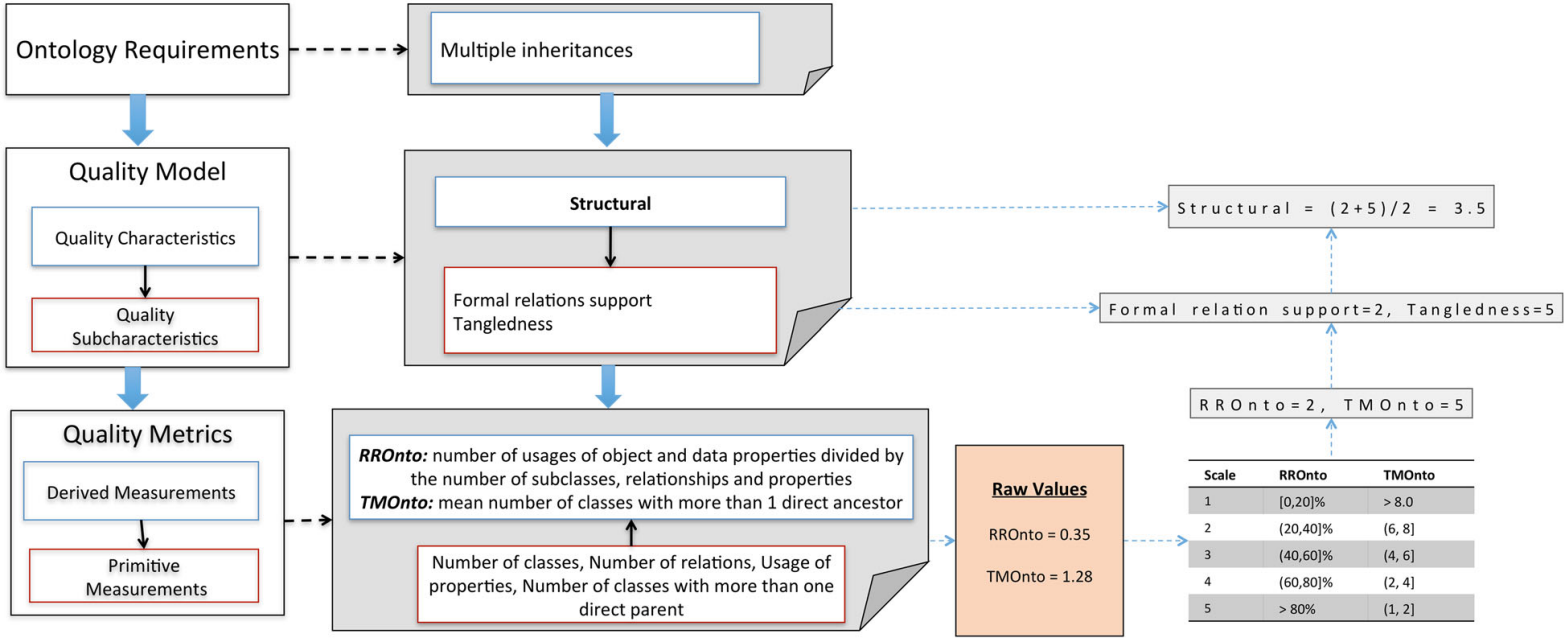
OQuaRE example that represents the traceability from the OQuaRE quality requirement to quality metrics divisions

Let us suppose that a user wants to evaluate the *ontology requirement* “Multiple inheritance of an ontology”, which might require to evaluate the “Structural” characteristic. This characteristic has 9 subcharacteristics, but only two will be used in this example (see Fig. 1) for simplicity, namely, “Tangledness” and “Formal relation support”. The traceability from the OQuaRE *quality metrics* to the *quality requirements* is shown in Fig. 1. “Tangledness” depends on the TMOnto metric, whose value depends on the mean number of classes with more than 1 direct ancestor, so two primitive measurements (number of classes and number of direct ancestors) are used for computing the raw value of the metric, which in this example is 1.28. Raw values are transformed into *quality scores* using a scaling function. The scaling method (see Table 4) is based on the recommendations and best practices of the Software Engineering community for software metrics and ontology evaluation metrics. For TMOnto, the scaling function transforms this value into the *quality score* 5 because the raw value is in the range [1, 2]. Given that “Tangledness” has only the TMOnto metric associated, this is also its score. In case one subcharacteristic has more than one metric associated, its score would be the weighted mean of the *quality scores* of the metrics. In Fig. 1 we can see that *quality score* for “Formal relation support” is 2, so the score of the “Structural” characteristic is 3.5, that is, (5+2)/2.

**Table 4:** OQuaRE static scale with [1-5] values, where 1 means not acceptable, 3 minimally acceptable and 5 exceeds the requirements

Metric\Score	1	2	3	4	5
LCOMOnto	>8	(6-8]	(4, 6]	(2, 4]	<=2
WMCOnto	>15	(11, 15]	(8, 11]	(5, 8]	<=5
DITOnto	>8	(6, 8]	(4, 6]	(2, 4]	[1, 2]
NACOnto	>8	(6, 8]	(4, 6]	(2, 4]	[1, 2]
NOCOnto	>12	(8, 12]	(6, 8]	(3, 6]	[1, 3]
CBOnto	>8	(6, 8]	(4, 6]	(2, 4]	[1, 2]
RFCOnto	>12	(8, 12]	(6, 8]	(3, 6]	[1, 3]
NOMOnto	>8	(6, 8]	(4, 6]	(2, 4]	<= 2
RROnto	[0, 20] %	(20, 40] %	(40, 60] %	(60, 80] %	>80 %
AROnto	[0, 20] %	(20, 40] %	(40, 60] %	(60, 80] %	>80 %
INROnto	[0, 20] %	(20, 40] %	(40, 60] %	(60, 80] %	>80 %
CROnto	[0, 20] %	(20, 40] %	(40, 60] %	(60, 80] %	>80 %
ANOnto	[0, 20] %	(20, 40] %	(40, 60] %	(60, 80] %	>80 %
TMOnto	>8	(6, 8]	(4, 6]	(2, 4]	(1, 2]

Those metrics adapted from object oriented programming have been scaled based on the best practices for object oriented programming and the metrics whose result is a relative value are scaled in percentage

### Adapting OQuaRE for ontology evolution

#### Definitions

In this section, we define a series of concepts related to ontology evolution from the OQuaRE perspective.

##### **Definition 1**

Versioned corpus of an ontology (*v* *C*_*θ*_): is a list of versions {*v*_*i*_} of the same ontology *θ*, where *i* represents the chronological position of *v*_*i*_ in *v* *C*_*θ*_.

The comparison of different versions of the same ontology highlights changes and commonalities between the versions [5]. The comparison can be done using metrics of different nature (real-valued metrics, factor, ordered factors, etc.). In order to include all of them in a common context, the method requires the adaptation of the metrics, because they need to satisfy the constraints described in Definition 2.

##### **Definition 2**

Comparison criteria (*f*_*θ*_): is a discretisation framework that, for every version *v*_*i*_∈*v* *C*_*θ*_, provides a vector *s*_*i*_ of integers that can be used to rank those versions in *v* *C*_*θ*_.

The number of components of the vector *s*_*i*_ is *r*. For example, if we use TMOnto as a unique *comparison criterion*, *f*_*θ*_ discretises its real-value, using the *quality score*, to the range [1,5]. Moreover, in this case these integers are related to the different qualitative levels defined by OQuaRE, although different levels could be used. Then, given two versions *v*_*i*_ and *v*_*j*_, if *f*_*θ*_ produces the scores 5 and 1 respectively, that means that *v*_*j*_ is more tangled than *v*_*i*_. Similarly, the remaining 13 metrics can be added to the *comparison criteria*, and this is what we propose as a means to analyse the evolution of ontologies. Therefore, the application of *f*_*θ*_ to *v*_*i*_ generates a vector *s*_*i*_ of 14 components. The more components the vector *s*_*i*_ has, the harder it is to compare and interpret the changes. For this reason we provide the user with some definitions whose aim is to describe different types of changes. Hence, given two consecutive versions *v*_*i*−1_,*v*_*i*_∈*v* *C*_*θ*_, with *i*>1, and given the vectors *s*_*i*−1_ and *s*_*i*_ obtained by the application of the comparison criteria *f*_*θ*_, a change in scale of version *v*_*i*_ from version *v*_*i*−1_ is described in Definition 3.

##### **Definition 3**

Change in scale: vector of change associated with different values of the components of the vector *s*_*i*_ with respect to *s*_*i*−1_. The vector *l*_*i*_, which is calculated as *s*_*i*_−*s*_*i*−1_, represents the levels in size and direction of the changes from *v*_*i*−1_ to *v*_*i*_ version, with *i*>1.

It should be pointed out that the *change in scale* applies to all the versions of an ontology except to the first one, which corresponds to *i*=1 in *v* *C*_*θ*_. Since the OQuaRE *quality scores* are the *comparison criteria* the *level* ranges from [-4, 4], so the direction can be positive or negative. For example, let us suppose a *v* *C*_*θ*_ that contains six elements *v*_1_, …, *v*_6_. The application of *f*_*θ*_ to *v* *C*_*θ*_ generates a matrix with 6 rows, like the one shown in Expression 1. The row *i* represents the vector *s*_*i*_ and has 14 components, with *i*=1,…,6.

1 $$ \begin{aligned} &\quad \; 1 \quad \ldots \;\;\ldots \;\;\ldots \;\;\,\ldots \;\; 14(r)\\ \begin{array}{c} s_{1}\\ s_{2}\\ s_{3}\\ s_{4}\\ s_{5}\\ s_{6}\\ \end{array} &\left(\begin{array}{lllllll} 5 &\quad 4 &\quad 2 &\quad 1 &\quad \ldots &\quad.&\\ 5 &\quad 4 &\quad 2 &\quad 1 &\quad \ldots &\quad.&\\ 4 &\quad 3 &\quad 2 &\quad 1 &\quad \ldots &\quad.&\\ 3 &\quad 4 &\quad 5 &\quad 1 &\quad \ldots &\quad.&\\ 1 &\quad 5 &\quad 5 &\quad 2 &\quad \ldots &\quad.&\\ 5 &\quad 1 &\quad 4 &\quad 3 &\quad \ldots &\quad.&\\ \end{array}\right) \end{aligned}  $$

Using as input the matrix in Expression 1 we apply the Definition 3 and obtain a matrix with 5 rows, like the one shown in Expression 2. The row *i* represents the change in scale by the vector *l*_*i*_, with *i*=2,…,6. In the context of *quality scores*, a negative component in *l*_*i*_ represents a decreasing level in the corresponding *quality score* of *v*_*i*_ from *v*_*i*−1_, a positive one means the opposite and 0 indicates that the metric score remains invariant.

2 $$ \begin{aligned} &\quad \;\; 1 \quad \; \ldots \quad \ldots \;\;\ldots \;\;\;\ldots \quad14 (r)\\ \begin{array}{c} l_{2}\\ l_{3}\\ l_{4}\\ l_{5}\\ l_{6}\\ \end{array} &\left(\begin{array}{rrrrrrr} 0 &\quad 0 &\quad 0 &\quad 0 &\quad \ldots &\quad. &\\ -1 &\quad -1 &\quad 0 &\quad 0 &\quad \ldots &\quad. &\\ -1 &\quad 1 &\quad 3 &\quad 0 &\quad \ldots &\quad.& \\ -1 &\quad 0 &\quad 0 &\quad 1 &\quad \ldots &\quad. &\\ 4 &\quad -4 &\quad -1 &\quad 1 &\quad \ldots &\quad.&\\ \end{array}\right) \end{aligned}  $$

We propose to use a summarised representation of the change in scale of the *r* metrics and between *v*_*i*_ and *v*_*i*−1_ by using the frequency distribution *F*_*i*_ associated with the change in scale *l*_*i*_, which is defined in the following way:

##### **Definition 4**

Frequency distribution of the chase in scale (*F*_*i*_): it is an ordered list of the frequencies *f*_*l*_ associated with the different change levels *l* in the vector *l*_*i*_.

The change levels range between *l*_*min*_ and *l*_*max*_. In the context of OQuaRE *quality scores*, *l*_*min*_ and *l*_*max*_ are −4 and 4 respectively. Therefore, in this case the frequency distribution *F*_*i*_ has 9 components, which represent the frequencies *f*_*l*_ of the ranks *l* from -4 to 4. For example, Expression 3 shows the frequency distributions of our running example. The interpretation of *F*_2_ is: there are 4 out of *r* metrics that have not suffered any change in scale between *v*_1_ and *v*_2_. The change is larger between *v*_2_ and *v*_3_ (*F*_3_) as there are 2 metrics that have decreased one scale and other 2 remain unchanged.

3 $$ \begin{aligned} & \quad \, f_{-4} \;\;\, f_{-3} \;\; f_{-2} \;\; f_{-1} \;\;\; \, f_{0} \quad \, f_{1} \quad \, f_{2} \quad \, f_{3} \quad \, f_{4}\\ \begin{array}{c}F_{2}\\ F_{3} \\ F_{4}\\ F_{5} \\ F_{6}\end{array} &\left(\begin{array}{lllllllll} \quad 0 & \quad 0 & \quad 0 & \quad 0 & \quad 4 & \quad 0 & \quad 0 & \quad 0 & \quad 0 \\ \quad 0 & \quad 0 & \quad 0 & \quad 2 & \quad 2 & \quad 0 & \quad 0 & \quad 0 & \quad 0 \\ \quad 0 & \quad 0 & \quad 0 & \quad 1 & \quad 1 & \quad 1 & \quad 0 & \quad 1 & \quad 0 \\ \quad 0 & \quad 0 & \quad 0 & \quad 1 & \quad 2 & \quad 1 & \quad 0 & \quad 0 & \quad 0 \\ \quad 1 & \quad 0 & \quad 0 & \quad 1 & \quad 0 & \quad 1 & \quad 0 & \quad 0 & \quad 1 \end{array}\right) \end{aligned}  $$

Hence the frequency distribution *F*_*i*_ can be used for describing different types of changes between two consecutive versions *v*_*i*−1_ and *v*_*i*_ with respect to the set of OQuaRE *quality scores*. Next, we define some associated statistics such as weighted means.

##### **Definition 5**

Forward Mean Change: weighted mean of the positive change levels *l*, calculated as:

$$\frac{\sum_{1}^{l_{max}} l\times f_{l}} {\sum_{1}^{l_{max}} f_{l}} $$

##### **Definition 6**

Backward Mean Change: weighted mean of the negative change levels *l*, calculated as:

$$\frac{\sum_{l_{min}}^{-1} l\times f_{l}} {\sum_{l_{min}}^{-1} f_{l}} $$

To avoid possible undefined values of the forward or backward means, we also use the size of the forward and backward changes defined as the numerator of the previous definitions, but considering absolute values |*l*| in backward mean changes. Now, Definition 7 provides the definition for the global mean change.

##### **Definition 7**

Mean change: weighted mean of the change levels *l*, calculated as:

$$\frac{\sum_{l_{min}}^{l_{max}} l\times f_{l}} {\sum_{l_{min}}^{l_{max}} f_{l}} $$

In our running example, the frequency distribution *F*_3_ does not provide a determined finite value for the forward mean change, whereas the backward mean change is −1 and the mean change is −0.5. The sizes of the forward and backward changes are 0 and 2, respectively.

The value of the *mean change* can be interpreted as follows:

- It takes a positive value when the forward mean change is greater than the backward one and negative when the opposite.
- It becomes zero when forward and backward mean changes take equal and finite values.
- It becomes zero if *v*_*i*_ and *v*_*i*−1_ are identical. In this case forward and backward mean changes do not take a determined finite value (undefined value).

The *mean change* provides information about changes in *quality scores*. For analysing the number of metrics that have changed regardless of the direction of the change, we define next the concept *magnitude of change*.

##### **Definition 8**

Magnitude of change: percentage of metrics with change in scale, which is calculated as follows:

$$\frac{\sum_{l\neq 0} f_{l}} {\sum_{l_{min}}^{l_{max}} f_{l}} $$

In our example, the *magnitude of change* of version *v*_2_ is 50 *%*. The largest number of metrics with changes happens in *v*_6_ (see *F*_6_ in Expression 3), having a *magnitude of change* of 100 %, but the *mean change* is 0.0. The major increase in *quality scores* happens in *v*_4_ (see *F*_4_ in Expression 3) with *mean change* 0.75.

#### A dynamic scaling function for ontology evolution

**Fig. 2:**
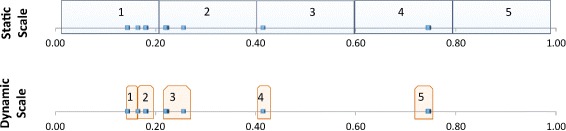
Example of the static and dynamic scale for RROnto metric. The x-axis represent the observer raw values of the metric for a *vC*. Semi-transparent rectangles shows the limits of the levels of the scale. While the static scale remains constant, the dynamic will depends of the observer raw values of RROnto in a *vC*

We propose to take advantage of the information available in the *v* *C*_*θ*_ to derive a dynamic scaling function. For this purpose, each ontology in such a corpus is processed with OQuaRE, so calculating the raw values of the 14 *quality metrics*. These original values are used for generating a scale in *k* categories determined by k-means clustering [25], which groups similar values into the same category by minimising the intra-class variance and emphasises the differences among categories maximising the inter-class variance. In this paper, the number of categories is *k*=5 because the OQuaRE scale is [1,5]. This is illustrated using Fig. 2. The metric RROnto measures the richness of relations and it is calculated using the mean number of usages of object and data properties divided by the number of subClassOf relationships and object properties. The standard scale for RROnto is shown in Table 4.

**Fig. 3:**
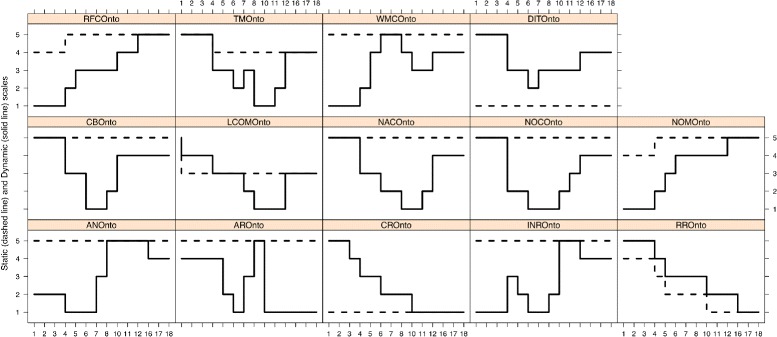
Graphical representation of the static and dynamic scaled metrics along the versions

The RROnto raw values obtained for all the versions within a *v* *C*_*θ*_ are represented in the x-axis of Fig. 2. The static scale is represented in the upper-part of the figure, and the dynamic scale obtained using k-means is shown in the bottom-part. While the raw RROnto value 0.74 is matched with the *quality score* 4 in the static scale, it is matched with 5 in the dynamic scale. It should be pointed out that the dynamic scale forces data to be categorised between 1 and 5, 1 being the lowest raw value found in *v* *C*_*θ*_ and 5 the highest. If the amount of different data is not enough to generate 5 categories the algorithm does not include any value in the lowest categories of the scaling function (see for example the solid line for DITOnto metric in Fig. 3). Therefore, the application of the dynamic scale should help users to study the evolution of the observed quality metrics values for all the versions within a *v* *C*_*θ*_.

### The ontology of Bioinformatics operations, types of data, formats, and topics (EDAM)

We are going to study the evolution of the EDAM ontology [21, 26]. The EDAM is an ontology of well established and familiar concepts that are prevalent within bioinformatics. The EDAM includes types of data, data identifiers, data formats, operations and topics. We have chosen this ontology as an example because:

- It is well documented and its developers use a control version system (CVS) [27] so that we can trace changes.
- Its source files are accessible online. The latest version (v1.9) is published in the official project web page. Links to old versions can be found in BioPortal (18 versions) and in the CVS (13 versions).
- It has received 900 mean visits per month since Oct-2013 to Apr-2014 and 6 declared projects use the EDAM.
- The number of versions (18) makes it an ontology of interest for studying its evolution. Its size (2 597 classes as mean) is intermediate, which facilitates the analysis of the results in this first application of the method.

## Results and discussion

### Experimental setup

The versioned corpus comprised the 18 EDAM versions in BioPortal as CVS content, which was processed using a software tool developed in house that implements the OQuaRE framework. This framework and tool are publicly accessible at http://sele.inf.um.es/oquare as a web form and a web service. The framework uses the OWL API [28] and Neo4j [29] for the calculation of OQuaRE metrics. We carried out the computation of the dynamic scaling by using the function bin.var of the package RcmdrMisc of R [30].

We applied a normalisation process to the 18 versions. In the normalisation, we removed deprecated classes and checked the consistency of the ontology. Before applying the normalisation, 4 out of 18 versions were discarded by the tool: one could not be processed by the OWL API, and the other three were found to be inconsistent by the reasoner Hermit [31]. Therefore, the versioned corpus contained 14 ontologies. In the remainder of this paper, we label each version according to its original id version. It should be pointed out that the statistics of change of a certain version *v*_*i*_ were calculated with respect to the previous processed version. For example, the *change* in *v*_16_ was calculated with respect to *v*_12_ because *v*_13_, *v*_14_ and *v*_15_ could not be processed.

The normalisation process made consistent *v*_13_ and *v*_14_ and, therefore, they were included in the study. We decided to perform two types of experiment: one with the deprecated classes (14 consistent ontologies) and one without the deprecated classes (16 consistent ontologies) with the goal of studying the impact of the obsolete classes in the structure of the ontology. We applied the tool to obtain the scores of the metrics, subcharacteristics and characteristics for all the versions. Such measurements were the *comparison criteria*, which allowed the scores to be obtained by using both the static scaling function and the dynamic one. After presenting those results, we will discuss the evolution of the EDAM in terms of *quality scores* and analyse the advantages and disadvantages of both scaling methods. The whole set of results is available at http://miuras.inf.um.es/oquare/jbsm2016.

### Analysis of quality characteristics with the static scale

Table 5 shows the results obtained at the quality characteristics level. Two *quality scores* are shown for each quality characteristic: original (org) and normalised (nrm). Bold numbers highlight *changes in scale*. Next, we discuss the changes observed in the quality characteristics.

**Table 5:** OQuaRE characteristics metric values for eighteen versions of the EDAM ontology

V.	Date	Status	Struct.		F. Adeq.		Reliab.		Operab.		Compat.		Maint.		Transf.		Mean	
			Org.	Nrm.	Org.	Nrm.	Org.	Nrm.	Org.	Nrm.	Org.	Nrm.	Org.	Nrm.	Org.	Nrm.	Org.	Nrm.
1	**2010-05-14**	beta	4.67	4.67	4.61	4.61	3.25	3.25	3.83	3.83	3.75	3.75	4.10	4.10	3.75	3.75	3.99	3.99
2	**2010-05-28**	beta	4.50	4.50	4.60	4.60	**2.88**	**2.88**	3,67	3.67	3.75	3.75	3.99	3.99	3.75	3.75	3.88	3.88
3	**2010-08-18**	beta	4,50	4.50	4.60	4.60	2.88	2.88	3.67	3.67	3.75	3.75	3.99	3.99	3.75	3.75	3.88	3.88
4	**2010-10-07**	beta	4,50	4.50	4.60	4.60	2.88	2.88	3.67	3.67	3.75	3.75	3.99	3.99	3.75	3.75	3.88	3.88
5	**2010-12-01**	beta	4.17	4.17	4.46	4.46	2.75	2.75	**4.00**	**4.00**	**4.00**	**4.00**	**4.23**	**4.23**	**4.00**	**4.00**	3.94	3.94
6	**2011-01-22**	beta	4.00	4.00	4.28	4.28	2.75	2.75	4.00	4.00	4.00	4.00	4.23	4.23	4.00	4.00	3.90	3.90
7	**2011-06-17**	beta	4.00	4.00	4.28	4.28	2.75	2.75	4.00	4.00	4.00	4.00	4.23	4.23	4.00	4.00	3.90	3.90
8	**2011-12-05**	beta	4.00	**3.83**	4.28	4.27	2.75	2.38	4.00	**3.83**	4.00	4.00	4.23	4.12	4.00	4.00	3.90	3.78
10	**2012-12-10**	beta	4.00	3.83	4.28	4.27	2.75	2.38	4.00	3.83	4.00	4.00	4.23	4.12	4.00	4.00	3.90	3.78
11	**2012-12-14**	release	**3.83**	3.83	4.11	4.27	2.75	2.38	4.00	3.83	4.00	4.00	4.23	4.12	4.00	4.00	3.85	3.78
12	**2014-02-18**	update	3.83	3.83	4.11	4.27	2.75	2.38	4.00	3.83	4.00	4.00	4.23	4.12	4.00	4.00	3.85	3.78
13	**2014-09-26**	update	-	3.83	-	4.27	-	2.38	-	3.83	-	4.00	-	4.12	-	4.00	-	3.78
14	**2014-11-14**	update	-	**4.00**	-	4.28	-	2.75	-	**4.00**	-	4.00	-	4.23	-	4.00	-	3.90
16	**2014-12-08**	update	3.83	4.00	4.11	4.28	2.75	2.75	4.00	4.00	4.00	4.00	4.23	4.23	4.00	4.00	3.85	3.90
17	**2014-12-16**	update	3.83	**3.83**	4.11	4.11	2.75	2.75	4.00	4.00	4.00	4.00	4.23	4.23	4.00	4.00	3.85	3.85
18	**2015-02-02**	update	3.83	3.83	4.11	4.11	2.75	2.75	4.00	4.00	4.00	4.00	4.23	4.23	4.00	4.00	3.85	3.85

These values are scaled from 1 to 5, where 1 is not acceptable and 5 exceeds the requirements. Bold numbers highlight *changes in scale* between two consecutive versions

We can observe in Table 5 that the mean *quality score* ranges from 3.99 in the first version to 3.85 in the last one, so its *quality scores* have always stayed between 3 and 4. A *quality score* higher than 3 reveals that good ontological engineering principles have been applied by the EDAM developers. However, this difference has not produced a *change in scale* in global terms. Despite this fact, investigating why the *quality score* decreased is relevant because lower OQuaRE levels provide users with more fine grain information. For example, those decisions made during the construction or modification of large and complex ontologies may have collateral effects in their engineering, which may have different implications from a quality perspective. For example, reducing the usage of properties might benefit the maintainability of the ontology but fewer queries might be asked. Therefore, a lower value in OQuaRE metrics related to the usage of properties would contribute positively to the “Maintainability” of the ontology but negatively to the “Formal relations support”. Understanding how different changes influence different quality aspects is difficult to study if we use only the mean *quality score*. This is why the analysis at the level of characteristics, subcharacteristics and even metrics is recommended.

First, we describe which characteristics have *changes in scale*. The analysis of the evolution of *quality scores* of the characteristics (between the first version and last one) shows that 6 out of the 7 quality characteristics had a change in scale: 4 positive and 2 negative. In the remaining case, there was no *change in scale* for “Functional Adequacy”. The score of the “Reliability” characteristic decreased from 3 to 2 in *v*_2_; and the “Structural” one decreased from 4 to 3 in *v*_11_. The scores for “Operability”, “Compatibility”, “Maintainability” and “Transferability” increased from level 3 to 4 in *v*_5_. Moreover, the ontology has maintained the score at this level since then. This behaviour happened for all their associated sub-characteristics. The scores for the whole set of sub-characteristics can be found at http://miuras.inf.um.es/oquare/jbsm2016.

### Analysis of the quality metrics with the static scale

Next, we describe the changes observed at the level of *OQuaRE metrics* because this enables us to focus on concrete structural changes, which can help us to discuss and explain the variations obtained in higher levels. Figure 3 (dashed lines) shows the *quality scores* of the static scale for the 14 OQuaRE metrics. It can be observed that 9 OQuaRE metrics did not change for any version. The 5 metrics that have changed are LCOMOnto, NOMOnto, RFCOnto, TMOnto and RROnto. Next, we discuss the impact of the changes in these metrics at the level of OQuaRE characteristics and sub-characteristics.

- RROnto had 3 *changes in scale*. The first 2 changes were consecutive and due to the usage of properties, which decreased 86 % between *v*_4_ and *v*_6_. Refactoring towards a common set of properties can often be a sign of good ontology engineering practise, however the usage measures the number of times that a property is linked with an entity through an axiom. For example, while *v*_4_ defines 16 properties with 6 734 usages, *v*_5_ and *v*_6_ define the same number of properties but with 1 979 and 937 usages respectively. The usage of properties also decreased 8 % between *v*_10_ and *v*_11_. This variation is smaller than the previous one but, together with an unusual increase in the number of relations (18 %), it triggered the *change in scale* of RROnto. This increase in the number of relations is a consequence of a structural change in *v*_11_: deprecated classes were grouped as descendants of an ontology class in the first taxonomic level and this increased the number of relations.
- RFCOnto and NOMOnto had 1 *change in scale* growing from 4 to 5 in *v*_4_. This behaviour was also related to the usage of properties. However, for these metrics such a primitive metric influences positively the *quality score* because, in the case of NOMOnto, the lower the mean number of property usage per class is the easier the maintainability of the ontology is. This behaviour triggered the change in scale for the characteristics “Operability”, “Compatibility” and “Transferability” in *v*_5_.
- TMOnto measures the distribution of the parents in the ontology. 10 % of the classes had more than 1 direct parent in *v*_4_, while this value grew up to 24 % in *v*_5_. This metric has a negative effect across the ontology because of the multiple inheritance, although this might be needed to reflect some aspects within the ontology. This fact influenced the decrease in the “Tangledness” subcharacteristic, which also contributed to the decrease of the the “Structural” characteristic. However, for this metric this change did not trigger by itself a change in scale, which was produced in *v*_11_ with the collaboration of RROnto.
- LCOMOnto uses the number of paths in the ontology in its calculation and it suffered one *change in scale* in *v*_2_. This metric is used in the subcharacteristics “Cohesion”, “Knowledge reuse”, “Learnability ”, “Recoverability” and “Availability”. Moreover, this metric is the unique used to measure “Cohesion” and “Availability”, so it has a deeper impact for these two subcharacteristics than for the others. On the one hand the lowest score for the “Structural” characteristic was for “Cohesion” but this did not trigger a change in scale for *v*_2_. On the other hand, the “Recoverability” and “Availability” are grouped in the “Reliability” characteristics and for it, the behaviour of the LCOMOnto metric triggered the *change in scale* in *v*_2_.

### Influence of deprecated classes

The presence of deprecated classes grew from 3.51 % (*v*_1_) to 29.58 % (*v*_18_). Deprecated classes caused inconsistencies in *v*_13_ and *v*_14_. Table 5 shows that there were no significant changes at the characteristic level between the ontologies with (Org) and without the deprecated classes (Nrm), but some changes happened at the metric level. The change in the Structural characteristic with deprecated classes anticipated the drop of RROnto to *v*_11_, whereas it happened in *v*_17_ in the normalised version. Besides, LCOMOnto temporarily descended to score level 2 between *v*_8_ and *v*_13_ in the normalised version. This effect on LCOMOnto could not be appreciated in the ontologies with the deprecated classes. Deprecated classes remain in the ontology, so they are influencing the OQuaRE results. For example, RROnto uses the number of subClassOf relations in the denominator, to which deprecated classes (see Table 2) contribute. The removal of the deprecated classes had an impact on this metric, which produced this effect of anticipating or delaying *changes in scale*. Moreover, the scaling function cushioned smaller changes such as the one produced by LCOMOnto.

### Application of the dynamic scale

We have obtained a dynamic scale using the EDAM ontology versions composing the experimental *v* *C*_*θ*_. The values obtained after applying the k-means clustering are shown in Table 6. Moreover, Fig. 3 shows the evolution of the values of the metrics for both the static (dashed lines) and dynamic scales (solid lines). It can be seen that the dynamic scale is able to capture more changes in those values than the static one. This is an expected result as the [1,5] scale limits for each metric is derived from the raw values of the metrics for the different versions of the ontologies. This means that both scales reflect different aspects and, therefore, are complementary in helping to understand the engineering and the evolution of the ontologies. Next, we discuss how changes are detected by both scales.

**Table 6:** Coordinates of the dynamic scale obtained after applying the k-means algorithm using the versions of the EDAM within the experimental *vC*

Metric\Score	1	2	3	4	5
LCOMOnto	[5.646945, 5.782834]	[5.505317, 5.505317]	[5.158599, 5.190406]	[5.072177, 5.093400]	[3.874391, 4.109421]
WMCOnto	[4.123580, 4.176131]	[1.931285, 1.931285]	[1.536827, 1.559519]	[1.401986, 1.478964]	[1.334862, 1.347192]
DITOnto	[–, –]	[14, 14]	[13, 13]	[12, 12]	[11, 11]
NACOnto	[1.275837, 1.279578]	[1.261488, 1.264644]	[1.245146, 1.245352]	[1.228666, 1.230561]	[1.099615, 1.104098]
NOCOnto	[1.332252, 1.342622]	[1.276790, 1.286796]	[1.263569, 1.263569]	[1.229043, 1.230952]	[1.103604, 1.108706]
CBOnto	[1.602873, 1.637277]	[1.559101, 1.559101]	[1.404925, 1.456697]	[1.230693, 1.281911]	[1.143644, 1.152976]
RFCOnto	[4.364891, 4.383669]	[2.306886, 2.306886]	[1.900142, 2.022841]	[1.541327, 1.564187]	[1.438217, 1.475449]
NOMOnto	[3.068605, 3.115014]	[0.799515, 0.799515]	[0.3790453, 0.3790453]	[0.2754958, 0.3078338]	[0.2071335, 0.2423935]
RROnto	[0.144421, 0.144694]	[0.164751, 0.180672]	[0.2195698, 0.2562910]	[0.4139807, 0.4139807]	[0.7441604, 0.7459092]
AROnto	[4.14, 5.00]	[7.0, 7.0]	[14.0, 14.0]	[16.0, 16.0]	[21.0, 21.0]
INROnto	[1.037050, 1.061705]	[1.094152, 1.099919]	[1.13177, 1.13177]	[1.227018, 1.228871]	[1.261331, 1.277758]
CROnto	[0.0, 0.0]	[0.35285 *e* ^−3^, 0.36778 *e* ^−3^]	[0.40420 *e* ^−3^, 0.40453 *e* ^−3^]	[0.45433 *e* ^−3^, 0.45433 *e* ^−3^]	[0.47103 *e* ^−3^, 0.48123 *e* ^−3^]
ANOnto	[1.097413, 1.102329]	[1.114493, 1.117287]	[1.131656, 1.131656]	[1.144306, 1.144975]	[1.150423, 1.153622]
TMOnto	[0.2556087, 0.2599199]	[0.2461048, 0.247064]	[0.2400970, 0.2436178]	[0.2171334, 0.2173766]	[0.09961501, 0.10456901]

The changes in some metrics were detected by both scales. In the case of RROnto, although the first version starts in 4 for the static scale and in 5 for the dynamic scale, both scales detected changes between the same pairs of versions, except for *v*_17_. However, this did not happen for RFCOnto, TMOnto, NOMOnto or LCOMOnto. The dynamic scale is more sensitive so it detected more changes between pairs of versions for these 4 characteristics. The static scale did not detect changes for nine metrics, but the dynamic one did. For example, while the DITOnto value remained in 1 in the static scale, in the dynamic scale it started in 5 and ended in 4. Moreover, it decreased to 2 in *v*_7_.

The value of DITOnto remained in 1 with the static scale for all the versions of the EDAM ontology. DITOnto measures the depth of the ontology. The raw values obtained for our corpus were (11, 11, 11, 11, 13, 13, 14, 13, 13, 13, 13, 12, 12, 12). All of them are greater than 8, which is scaled to the *quality score* 1, according to the best practice applied. However, in the field of ontologies an appropriate value for DITOnto might depend on many factors, and it is here where the dynamic scale can complement the static one. According to [32], well-structured OO systems have a forest of classes rather than one large inheritance lattice. However, whether a high or low value is desired from a metric for better code quality still must exercise judgement when determining the best approach for the task at hand. According to [32], the lower the DITOnto the better, so the OQuaRE scaling method matches DITOnto “low values” to 5 and “high values” to 1. Then, the dynamic scale uses the lowest and highest values observed for the versions of the ontology to assign the scores 5 and 1, respectively. With this scale, the highest *quality scores* were reached from *v*_1_- *v*_4_, then it went down from *v*_4_−*v*_5_ and again from *v*_6_- *v*_7_, then it remained stable until *v*_12_, where it again increased one level. As we have explained previously, it should be pointed out that the dynamic scaling method for DITOnto did not span the range [1 …5] because there were only 4 raw values observed.

### Analysis of major changes between versions

**Fig. 4:**
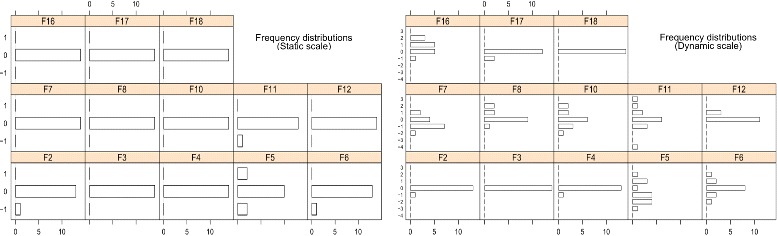
Frecuency distributions of the changes in scale

The graphical representation of the frequency distributions *F*_*i*_ is shown in Fig. 4. The left-half of the Figure shows the frequency distributions *F*_*i*_ obtained with the static scale, on the right-half the ones obtained with the dynamic scale. For each box, the y-axis represents the components from the levels *l*_*min*_ to *l*_*max*_; it should be pointed out that this figure just represents in the y-axis those components with at least one observed frequency *f*_*l*_ distinct than 0 for any version in *v* *C*_*θ*_). Finally, the x-axis represents the frequency of each component. For example, with the static scale and for *v*_8_ (*F*_*i*_=8) the frequency of *l*_0_ is 13 because the value of 13 metrics did not *change in scale* with respect to the previous version; similarly, with the dynamic scale and for *v*_2_ (*F*_2_) the frequency of *l*_−1_ is 1 because 1 metric (LCOMOnto) decreased one level.

Now we describe how to use the *magnitude* and *mean change* to analyse major changes between consecutive versions. This will be done by discussing the data shown in Table 7, where rows 2–5 show the values of the four statistics of change using the static scale, and rows 6–9 show those statistics for the dynamic scale.

**Table 7:** Statistics for static and dynamic scales: *magnitude of change*, *mean change forward*, *mean change backward*, and *mean change*

	Change	v2	v3	v4	v5	v6	v7	v8	v10	v11	v12	v16	v17	v18
Sta.	Magnitude	7 %	0 %	0 %	28 %	7 %	0 %	0 %	0 %	7 %	0 %	0 %	0 %	0 %
	Mean. For	1	-	-	1	-	-	-	-	-	-	-	-	-
	Mean. Back	-	-	-	1	1	-	-	-	1	-	-	-	-
	Mean	-0.07	0.00	0.00	0.00	-0.07	0.00	0.00	0.00	-0.07	0.00	0.00	0.00	0.00
Dyn.	Magnitude	7 %	0 %	7 %	79 %	42 %	71 %	36 %	56 %	56 %	21 %	64 %	14 %	0 %
	Mean. For	-	-	-	1.25	1.33	1.00	1.50	1.50	1.75	1.00	1.38	-	-
	Mean. Back	1.00	-	1.00	1.67	1.33	1.12	1.00	1.25	1.75	-	1.00	1.00	-
	Mean	-0.07	0.00	-0.07	-0.71	0.00	-0.50	0.36	0.07	0.00	0.21	0.71	-0.14	0.00

The symbol “-” in this table represents the undefined value

#### Analysis of magnitude of change

The *magnitude of change* with the static scale was different than 0 for *v*_2_, *v*_5_, *v*_6_ and *v*_11_ (see Table 7 row 2). For example, the largest *magnitude* of change happened for *v*_5_, 28 %, and this was a consequence of the changes in RFCOnto, NOMOnto, TMOnto and RROnto; these changes in the OQuaRE *quality metrics* can be observed in Fig. 3 (dashed lines). For *v*_2_, *v*_6_ and *v*_11_, the *magnitude of the change* is 7 % because only one metric had a change of level. There were no changes in the *quality scores* for the rest of the versions. The *magnitude of change* with the dynamic scale was different than 0 for 11 out of 13 versions. This is a consequence of the higher sensitivity of the dynamic scale. This scale enabled the identification of versions like *v*_3_ or *v*_18_ to be very similar with respect to their previous one, because the *magnitude* and *mean change* were 0 % and 0.00 respectively. By similar we mean that there were not enough changes between them that produced a *change in scale* for any of the OQuaRE metrics.

In order to analyse pairs of consecutive versions, we are going to use the median (*M*_*e*_) of the absolute difference between the values of the 14 metrics, and the Wilcoxon test for contrasting the alternative hypothesis *M*_*e*_>0. Table 8 sorts the versions by increasing critical value and *p*-value associated with the null hypothesis (*M*_*e*_=0) for each test performed. These results show that:

- We reject the null hypothesis (*M*_*e*_=0) in all the comparisons, so we can interpret that all the changes are significant.

**Table 8:** Versions sorted from less to high critical value and *p*-value associated with the null hypothesis *M*_*e*_ after applying the test of Wilcoxon using the difference in absolute values of the median of 14 OQuaRE metrics and consecutive pairs of versions

Version	Critical_value	*P*_value
18	0.0001825782	1.263087 *e* ^−3^
2	0.0013102421	1.263087 *e* ^−3^
3	0.0020865871	1.263087 *e* ^−3^
4	0.0021207897	1.263087 *e* ^−3^
17	0.0044867447	1.263087 *e* ^−3^
8	0.0072293707	6.103516 *e* ^−5^
12	0.0113504041	1.263087 *e* ^−3^
10	0.0119625746	8.308472 *e* ^−4^
6	0.0259642025	8.308472 *e* ^−4^
11	0.0303236313	8.308472 *e* ^−4^
16	0.0324480617	8.308472 *e* ^−4^
7	0.0420278822	6.103516 *e* ^−5^
5	0.1587347761	8.308472 *e* ^−4^
- We have evaluated the *magnitude of change* using the *quality scores* (scaled metrics). The critical value shows the magnitude from which the difference median (*M*_*e*_) is significantly higher at the 0.05 level of significance. Using this criterion for sorting the changes between versions we obtain that the largest change happens in *v*_5_.
- The four versions with the largest changes according to this analysis are also the four versions with the highest *magnitude of change* for the dynamic scale, as shown in Table 7 row “Magnitude”. This shows the goodness of the criteria used in the dynamic scaling function.

#### Analysis of mean changes

The *mean change* using the static scale is negative because the score of one metric decreased for *v*_2_, *v*_6_ and *v*_11_ (see Table 7 row 6). However, the *magnitude of change* had a different evolution. The largest *magnitude* happened for *v*_5_, but the mean change for *v*_5_ was 0.0, because the number of positive weighted changes was equal to the number of negative ones. For this particular case, two metrics increased 1 level (RFCOnto and NOMOnto) and 2 exactly the opposite (TMOnto and RROnto) (see dashed line in Fig.3). The higher sensitivity of the dynamic scale is also observed in the *mean change* values, because more changes were detected. For example, if we focus on *v*_5_, the “Mean. Back” (1.67) was higher than the “Mean. For” (1.25) regardless of the number of metrics that had changed. Therefore, the “Mean” is -0.71, so there were more negative changes than positive ones.

**Fig. 5:**
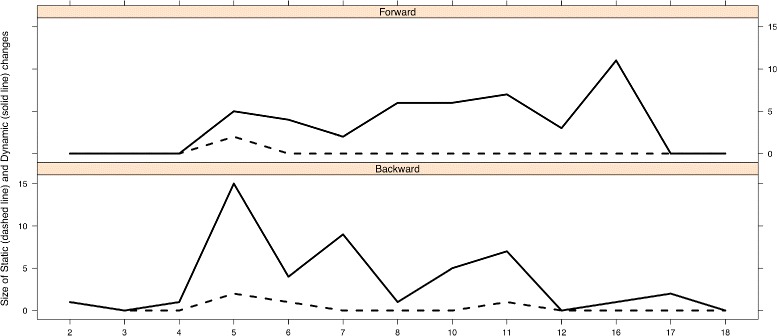
Statistics of size for static and dynamic scales: *forward mean change size* and *backward mean change size*

As a complement, the graphical representation of the *backward* and *forward mean change size* is shown in Fig. 5. The upper-half of this figure (“size forward”) stands for the positive changes, whereas the lower-half (“size back”) represents the negative ones. The largest positive change happened for version *v*_16_, and the largest negative one was for *v*_5_.

### Profile of change in *quality scores*

**Fig. 6:**
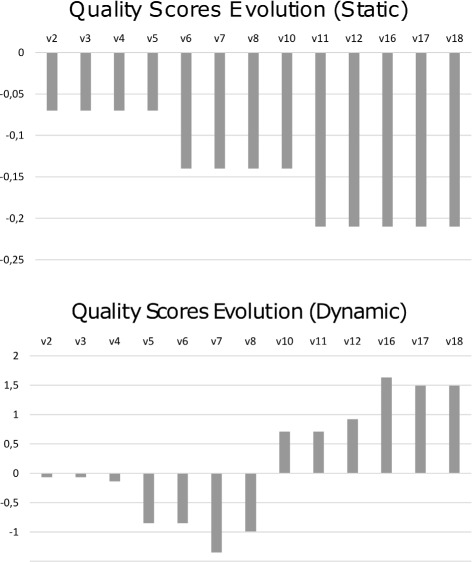
Graphical representation of the accumulative *mean change* using the static and dynamic scales
- The complementary use of the dynamic scale allows a different evolution to be observed. The mean change for the first 7 versions was negative, whereas it was positive for the next 9 versions. As a consequence, the accumulative mean change growed from -1,35 to 1,63. Finally, it decreased until 1.49 for *v*_17_ and remained constant for *v*_18_.

Regardless of the scale used, the information provided by the *mean change* can be used to calculate a profile of quality based on the OQuaRE framework. This profile takes into account the accumulative *mean changes* during the whole life-cycle of the ontology. Figure 6 shows the evolution of the *quality scores* using both scales:

- The use of the static scale shows a trend of negative *mean change*. The accumulative *mean change* value remained negative for all the versions and all the pairs, which is also reflected in the decrease of the *quality scores* of the characteristics as mean from 3.99 to 3.85, which was discussed previously.

Finally, if we take into account the status used to define each version in BioPortal: they are considered beta from version 1 to 10. Using the dynamic scale, we observe that the *quality scores* decreased until *v*_7_, and in particular in *v*_5_ with the lowest *mean change* (see Fig. 6). Having such changes during the beta stage makes sense. Once the ontology is considered released, the increase of the *quality scores* was over the mean.

### Relation between quality scores and the level of activity in an ontology

So far, we have analysed aspects related to variability in the *quality scores*. Now, we study the possible relation between these changes and the level of activity in an ontology. The *level of activity* has been measured in [3] in terms of changes in ontology classes, namely, number of classes that have been added, deleted or modified. These three variables are calculated by Bubastis [8], so we call them the *Bubastis variables*.

In http://miuras.inf.um.es/oquare/jbsm2016, several Principal Component Analysis (PCA) studies can be found. Here, we use the three statistics related to *mean change* (using the dynamic scale) and the *Bubastis variables* for performing a PCA, with the objective of obtaining the relation between these two different ontology aspects, as well as obtaining a bi-dimensional representation of the changes between two versions.

**Fig. 7:**
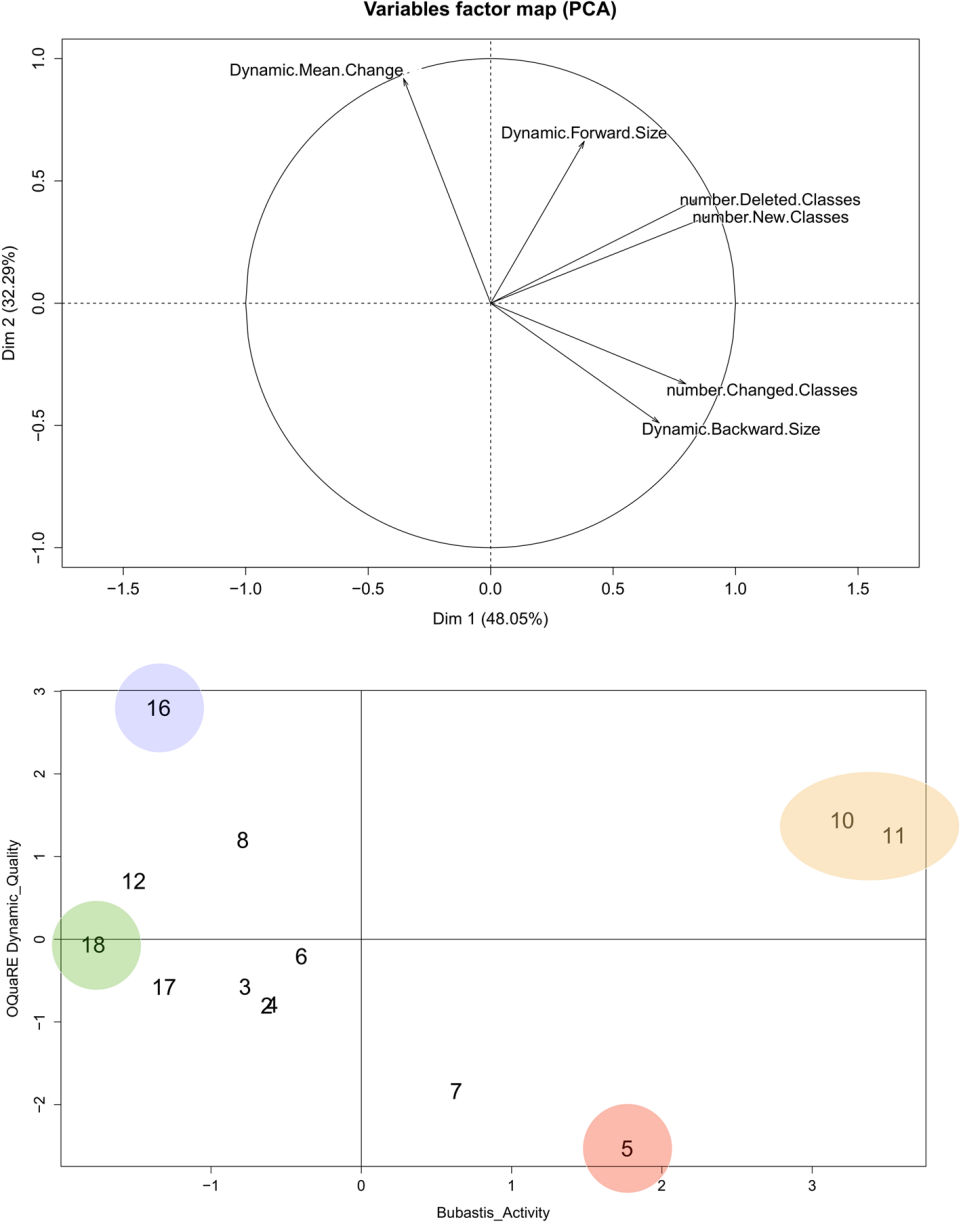
Principal Component Analysis: factors and principal components plots

The coordinates of the variables for the new axis are shown in Table 9, and they are graphically represented in Fig. 7 upper half. The variable representation of Fig. 7 suggests the presence of two normalised uncorrelated factors:

- The *Bubastis variables* have the largest positive correlations (0.88, 0.80 and 0.85, for ‘new’, ‘changed’ and ‘deleted’ classes respectively) with Factor 1 (represented in the x-axis), so we interpret this factor as a gradient representing the increasing volume of activity associated with the Bubastis activity. We call this factor *Bubastis Activity*.

**Table 9:** Representation in 2-dimensions of the coordinates of the variables for the new axis

	*(x-axis)* Factor 1	*(y-axis)* Factor 2
Number.New.Classes	0.8862	0.3539
Number.Changed.Classes	0.7970	0.3300
Number.Deleted.Classes	0.8458	0.4253
Dynamic.Backward.Size	0.6883	−0.4895
Dynamic.Forward.Size	0.3823	0.6623
Dynamic.Mean.Change	−0.3557	0.9186
Factor Name	Bubastis Activity	OQuaRE Dynamic Quality

Three statistics related to *mean change* (using the dynamic scale) and the *Bubastis variables* have been used for performing a PCA, with the objective of obtaining the relation between these two different ontology aspects. The variable representation of Fig. 7 suggests the presence of two normalised uncorrelated factors: *Bubastis Activity* and *OQuaRE Dynamic Quality*. The representation of these coordinates can be found in Fig. 7 above
- The Dynamic *mean change* has the largest positive correlation (0.92) with Factor 2 (represented in the y-axis), whereas dynamic backward size has a negative correlation with this factor. Those facts allow us to interpret this second factor as a gradient from lower OQuaRE *quality scores* to higher ones. We call this factor *OQuaRE Dynamic Quality*.

According to the previous comments, the versions represented in the first diagonal will be relevant in activity and quality, the more the farther from the origin they are.

The two previous factors explain more than 80 % of the information contained in the six variables shown in Table 9; and the first factor explains roughly 48 % of such information. Apart from the two factors, in Fig. 7 we also observe the next correlations using the Pearson test: (1) the number of classes deleted and new classes (0.99, *p*-value 0.0000); and (2) the dynamic mean change is almost independent of new (-0.01, *p*-value 0.9650) and deleted classes (0.07, *p*-value 0.8137) and (3) the dynamic forward size is almost independent of the number of changed classes (0.01, *p*-value 0.9808). Those pairs whose *p*-value is lower than 0.05 indicate a significant correlation.

Figure 7 bottom represents the principal components of the changes between consecutive versions in our *v* *C*_*θ*_, where four changes can be highlighted:

- The *Bubastis activity* of *v*_16_ was below the mean value. However, this activity produces a remarkable increment in the OQuaRE quality scores using the dynamic scale.
- The *Bubastis activity* of *v*_10_ and *v*_11_ was atypically high with respect to the rest of the versions. Moreover, the OQuaRE quality scores using the dynamic scale are over the mean value.
- The *Bubastis activity* of *v*_5_ was over the mean, producing a decrease in the OQuaRE quality scores using the dynamic scale and a high level in the number of classes changed.
- The *Bubastis activity* of *v*_18_ was the lowest and around the mean value in OQuaRE quality scores using the dynamic scale.

The most relevant changes obtained by this representation are the same as those obtained by the mean change statistics shown in Fig. 5, where *v*_5_ and *v*_16_ had the highest value of back and forward size respectively.

### A view on the evolution of the EDAM ontology

In this section we discuss how the application of our method enables some insights about the EDAM ontology and its evolution in terms of *quality scores* as well as the benefits of using the static or dynamic scales.

If we analyse the quality of the EDAM ontology from the OQuaRE perspective, we can identify different strengths and flaws, driving our attention to those *quality scores* obtained for the latest version analysed *v*_18_ (see Table 5). According to the OQuaRE static scale, the mean value 3.85 reveals that good ontological engineering principles have been applied. The analysis of the characteristics and sub-characteristics gives us more information. Next, we comment on the results for the highest and lowest score: maintainability, functional adequacy and reliability (4.23, 4.11 and 2.75 respectively).

- The highest *quality score* is obtained for maintainability (4.23). All its subcharacteristics associated have *quality score* over 4 (see values at http://miuras.inf.um.es/oquare/jbsm2016). This reveals some strengths of the EDAM, such as the reduced rate of negative side-effects due to changes in the ontology (modification stability 4.60) and the possibility to validate the ontology and detect flaws on it (testability 4.00).
- The second highest *quality score* applies to functional adequacy (4.11). For example, the EDAM is good for use as a controlled vocabulary to avoid heterogeneity of terms because all their classes have labels expressed in natural language. However, not all its subcharacteristics obtain high scores. For example, one weakness of the EDAM is elucidated by the score of the inference subcharacteristic. Its score is 1.0 due to the low usage of properties, despite the fact it is defined using a formal language. The absence of instances also contributed to this score.
- The lowest score is obtained for reliability (2.75), whose subcharacteristics are recoverability (2.50) and availability (3.00). The recoverability score is below 3, so it can be considered as a weakness of the EDAM because in case of inconsistency, incompleteness or redundancy of the content of the ontology, that would be difficult to re-established and to recover the ontology’s performance.

There is only another subcharacteristic with a *quality score* under 3, formal relation support, whose score is 1. The formal relations support measures the capability of the ontology to represent relations supported by formal theories different from taxonomy. This is calculated by analysing the usage of properties (RROnto). As we have shown in previous sections, RROnto has a score of 1 in the latest versions whereas the value of the first version was 4, which makes it a potential weakness of the ontology in the latest versions. The previous discussion about RROnto comes from the comparison of different versions, so it is done in terms of evolution. Continuing with the analysis of the evolution of the EDAM ontology from the OQuaRE perspective, we can draw the following conclusions:

- *v*_5_, *v*_2_, *v*_7_ and *v*_11_ were the versions with the highest *magnitude of change*, that is, number of metrics with changes. The analysis of the characteristics using the static scale has revealed that, as mean, there are no *changes in scale* in the EDAM ontology. This is also observed in the negative trend of the accumulative *mean change* when the static scale is used (Table 6). Interestingly, the dynamic scale has revealed the observation that the accumulative *mean change* trend is positive from *v*_7_ to *v*_18_.
- At the characteristics level, the application of the static scale to the EDAM ontology has revealed that the evolution of the ontology has produced higher quality scores for four characteristics, and lower ones for two of them, as can be observed in Table 1.
- The analysis of changes at the OQuaRE metrics level helps us to identify that the usage of properties is the reason that has triggered the major descend in *quality scores* between *v*_4_- *v*_6_, and again between *v*_10_−*v*_11_. Moreover, an unusual increment of the number of relations in *v*_11_ triggered this change in scale. It should be pointed out that the application of our method can draw out these types of suggestions.

### Discussion about the method

In the previous sections we have described the main results of our work, as well as provided some discussion about the application of the method to the EDAM. Next, we provide some discussion about different aspects of the method.

In our previous work, the application of the standard, static scaling function used by OQuaRE proved its usefulness to detect strengths and flaws of ontologies and even to detect changes between versions of the same ontology. However, we believed that the use of more precise and sensitive methods for detecting changes would allow OQuaRE to be more supportive of ontology evolution processes. This is why we have proposed the dynamic scaling function, which should be used in conjunction with the static one, because they provide complementary information. Hence, this does not mean that the static scaling function cannot be used on its own for ontology evolution. It can be used to measure how the different versions have changed across their history, taking into account fixed criteria. For example, here we have evaluated the EDAM using the static function using as reference the current configuration that evaluates the ontology from an engineering point of view. This static scaling approach enables users to measure the quality of ontologies using a common framework, but, of course, this framework can be extended or fit to certain contexts in case that the context is clearly identified. Nevertheless, the dynamic scaling function should provide more useful information for ontologies for which new versions are frequently released or that do not constitute major changes with respect to the previous ones.

The development a common reference framework that can be used for those different requirement scenarios is a challenging task. An open question is whether the ranges can be universally set for the static scaling method. The dynamic scaling function tries to overcome this uncertainty by performing an evaluation based on the behaviour of the ontology during its evolution. It should be pointed out that the goal of the *dynamic scale* is not to replace or substitute the static one. In fact, the dynamic function does not discretise the raw values of the metrics using a continuous function, but the limits are set on the observed values (see Fig. 2). However, the dynamic scale result could be used to define new profiles based on re-adjusted static scales.

As future work, we propose to use the lessons learned in this experiment to analyse a larger set of ontologies. From our experience, reaching a community agreement for certain aspects of ontologies is not always an easy task, such as to what extent axiomatic richness is needed in biomedical ontologies [33]. On the one hand, those biomedical ontologies used as simple plain taxonomies or controlled vocabularies do not need a complex axiomatisation. On the other hand, those biomedical ontologies used as domain ontologies should be as rigorous and axiomatically rich as possible.

This debate is also related to the OQuaRE quality model. For example, the static scaling of the metric NOMOnto (see Table 4) could be interpreted as favouring more plain taxonomies over heavily axiomatised ontologies, because it would not be very difficult for ontologies with low axiomatisation to obtain a high *quality score* for NOMOnto. Another example, ontologies without instances have lower scores for some metrics, but sometimes the absence of instances is a design criterion for such ontologies. In such cases, the metrics that take into account instances should not be applied, or not considered relevant.We are currently working on enabling OQuARE profiles, which would allow users or communities of users to customise the associations between OQuaRE metrics, subcharacteristics and characteristics. The future OQuaRE users will be able to include new metrics or to define the scaling functions. The new metrics will have to be associated with current sub-characteristics. This solution is useful for users and communities with particular needs.

We consider that we could extend the idea of the dynamic scale and obtain a repository-based scale by using a repository like Bioportal [2] or AberOWL [34] as reference. The repository-based scale would be the result of applying the dynamic scaling method proposed in this paper but considering a *v* *C*_*θ*_ where *θ* represents the ontologies and versions within the repository. This repository-based scale would provide users some feedback to determine the ranges of the static scaling function based on a large set of existing ontologies. However, working with large repositories that can contain hundreds or thousands of versions for some ontologies can be challenging. We plan to use a “sliding window” approach, which would include the last 10-20 versions of an ontology, or *x* versions that cover the whole life-cycle of the ontology and having them equally separated across the time period. Such representative sample of versions would be used for creating the dynamic scaling function. Finally, the inclusion of new unsupervised clustering algorithms that automatically decide the number of categories of *quality scores* for each metric based on the raw data is also in our future work.

## Conclusions

We have developed a method that combines the analysis of versions with an ontology quality evaluation framework. The main objective of this paper was to study how the OQuaRE framework can support ontology evolution processes by informing, from the perspective of ontology quality, about the changes observed across the different versions of an ontology.

The two scaling functions proposed in this work should be jointly used for a better understanding of the engineering and the evolution of an ontology. The static scale is more useful when a single version of an ontology needs to be inspected and evaluated from an engineering point of view, or when there are significant differences between consecutive versions. However, when the different versions of an ontology are less distinct and evolution-oriented studies are our goal, the dynamic scale is able to provide more information. If we assume that the scaling function normalises the values regardless of the type of scale used, the values can be grouped and compared as done in this work with the *magnitude of the change* or the *mean change* between versions. It should be noted that judging the evolution of an ontology in terms of how its content conforms to the domain that is to be represented by the ontology are beyond the scope of this work. That would be the main objective of complementary methods such as realism-based ones [35, 36].

The application of the method to the EDAM reveals that good ontological engineering principles were applied in its development. The analysis of changes in the *quality scores* at both subcharacteristic and metric levels have shown the capability of the OQuaRE framework to identify weaknesses and strengths of the ontology. The OQuaRE metrics are capable of identifying changes in the engineering of the different versions of the ontology. The design decisions of the developers of the ontology have produced 18 versions of the EDAM ontology, and we have been able to describe the impact of such decisions from the quality perspective provided by OQuaRE: the scores for four characteristics increased, one characteristic remained invariant, and the scores for two characteristics decreased. Furthermore, our study has found relations between the level of class activity and the variability of *quality scores* for the EDAM ontology. Evaluating the relation between these changes in the quality scores and the design decisions of the ontology developers is beyond the scope of the present work. Our method provides the developers with data they can use for evaluating whether their decisions have the expected impact on the quality scores of the ontology.

In summary, we believe that the OQuaRE framework contributes to the engineering of the analysis of the evolution of ontologies and that provides relevant information for developers about the evolution of their ontologies.

## Abbreviations

EDAM: ontology of bioinformatics operations, types of data, formats, and topics
OQuaRE: ontology quality requirements and evaluation
OO: object oriented
PCA: principal component analysis
SQuaRE: software product quality requirements and evaluation
